# Comparing apples and pears in studies on magnitude estimations

**DOI:** 10.3389/fpsyg.2013.00332

**Published:** 2013-06-18

**Authors:** Mirjam Ebersbach, Koen Luwel, Lieven Verschaffel

**Affiliations:** ^1^Department of Psychology, University of KasselKassel, Germany; ^2^Educational Research and Development, Hogeschool-Universiteit BrusselBrussel, Belgium; ^3^Faculty of Psychology and Educational Sciences, Katholieke University LeuvenLeuven, Belgium

**Keywords:** magnitude estimations, mental representation, number line, symbolic and non-symbolic magnitudes, estimation biases

## Abstract

The present article is concerned with studies on magnitude estimations that strived to uncover the underlying mental representation(s) of magnitudes. We point out a number of methodological differences and shortcomings that make it difficult drawing general conclusions. To solve this problem, we propose a taxonomy by which those studies could be classified, taking into account central methodological aspects of magnitude estimation tasks. Finally, we suggest perspectives for future research on magnitude estimations, which might abandon the hunt for the mathematical model that explains estimations best and turn, instead, to investigate the underlying principles of estimations (e.g., strategies) and ways of their improvement.

## Introduction

There is an ongoing debate among researchers concerned with magnitude estimations on how the relationship between subjective estimations and objective magnitudes may be described best. This issue is important as poor estimation performance – such as in number line estimation tasks – is associated with limited mathematical abilities in children (e.g., Booth and Siegler, 2008; Geary et al., 2008). Furthermore, the characteristics of the underlying mental representation of magnitudes including its systematic biases are often directly inferred from the estimations (e.g., Siegler and Opfer, 2003).

Initially, two fundamental models have been proposed on how magnitudes might be mentally represented. The logarithmic ruler model (Dehaene et al., 1990; Dehaene, 1997) assumes that magnitudes are represented with constant variability on a mental number line. However, representations of larger numbers are located closer to each other and thus overlap compared to smaller numbers. The accumulator model, in contrast, states that magnitudes are represented equidistantly but with proportionally increasing variability (i.e., scalar variability: Gibbon and Church, 1981; Whalen et al., 1999; Huntley-Fenner, 2001). Studies promoting the logarithmic model usually employed relative magnitude estimation tasks such as identifying the larger of two numbers (e.g., Dehaene et al., 1990), while other studies used absolute magnitude estimations that required the approximate transformation between two magnitudes (e.g., generating 23 key presses without counting: Whalen et al., 1999). Only absolute magnitude estimations will be considered further in this article as only these represent estimations in a narrower sense, that is, “a process of translating between alternative quantitative representations, at least one of which is inexact” (Siegler and Booth, 2005, p. 198).

Based on the initial accounts, Siegler and colleagues (e.g., Siegler and Opfer, 2003) investigated how the estimation pattern of magnitudes develops using the number line task. Participants usually mark the position of given numbers on a number line, ranging for instance from 0 to 100 or from 0 to 1000. It has been demonstrated that in young children and for relatively large number ranges, in particular, the estimation pattern exhibits a logarithmic shape, whereas for small number ranges and in older children and adults, the pattern is linear and quite exact, without scalar variability.

This research was the starting point for further studies aiming to explain typical biases in numerical estimations of children and adults. Alternative models to a logarithmic model with constant variability and a linear model with scalar variability have been proposed, that is, segmented linear models (e.g., Ebersbach et al., 2008; Moeller et al., 2009) or a cyclic power model (e.g., Barth and Paladino, 2011). Moreover, a simple power model, adopted from psychophysical research (e.g., Stevens, 1957), was put forward to describe systematic biases in adults' numerical estimations (e.g., Crollen et al., 2011). A debate has started and is still going on about which model is best suited to explain the relationship between estimations and actual magnitudes (e.g., Barth and Paladino, 2011; Opfer et al., 2011; Ashcraft and Moore, 2012; Bouwmeester and Verkoeijen, 2012).

In the present article, we aim at emphasizing that studies employing absolute magnitude estimations to investigate the characteristics of the mental representation of magnitudes are often hardly to compare as they involve a broad range of methodological approaches that apparently may lead to different outcomes concerning the shape, accuracy, or variability of the estimations. To address this problem, we propose a taxonomy by which many of the conducted (and future) studies may be classified, which might help to evaluate the comparability, reliability, and validity of studies. Finally, implications for future research will be suggested.

## Lacking comparability of the studies

Studies involving absolute magnitude estimations differ broadly with regard to the tasks, the stimuli, and the methods of analysis. Hence, even additional studies might provide no further clarity on children's and adults' estimation abilities and the nature of their underlying mental representations as long as apples and pears are collected into the same basket. In the following, we will give some examples that are directly related to the taxonomy proposed later.

First, estimations can be conceived as numerical or non-numerical (Siegler and Booth, 2005). Numerical estimations involve a magnitude in a symbolic format (i.e., a number word or a numeral) that has to be transferred approximately into another – symbolic or non-symbolic – magnitude (e.g., telling the number of dots), or vice versa. This type will be referred to as *symbolic* estimations in the following. Non-numerical – or *non-symbolic estimations*, in contrast, refer to the approximate transformation between two non-symbolic magnitudes (e.g., reproducing a number of dots by key presses). Crollen et al. (2011) showed that symbolic and non-symbolic estimations of adults differ both qualitatively and quantitatively. Symbolic estimations yielded typical biases – that is, under- or overestimations, respectively – that could be well described by a power function. Non-symbolic estimations (i.e., reproduction task), in contrast, were relatively accurate and were described best by a largely linear function.

The differences between symbolic and non-symbolic estimations might be explained by the assumption of format-dependent representations of magnitudes (e.g., Dehaene, 1992; Cohen Kadosh et al., 2011; Lyons et al., 2012; for a review see Cohen Kadosh et al., 2008), although a format-independent representation has been proposed, too (e.g., McCloskey et al., 1985; Barth et al., 2003; Walsh, 2003). Evidence for format-dependent representations comes from fMRI measures showing that different formats activate distinct brain regions (e.g., Vogel et al., 2013). Furthermore, Roggeman et al. (2007) provided evidence that (at least small) symbolic magnitudes are mentally represented by place codes, that is, as activation of a specific position on the mental number line, corresponding to the target magnitude. Non-symbolic magnitudes, in contrast, are represented by summation codes, that is, as activation of a whole segment of the number line up to the corresponding position of the target magnitude. Place codes reflect a local and thus more precise activation on the number line than summation codes and might thus explain a higher accuracy of symbolic compared to non-symbolic estimations. Furthermore, it has been assumed that different transformation paths exist between distinct representational codes (e.g., bi-directional mapping model: Castronovo and Seron, 2007) that might differently affect children's estimations, in particular, whose number knowledge is not fully developed yet. They might perform poorer in symbolic estimations that require the comprehension or production of number symbols, compared to non-symbolic estimations. Evidence for this assumption stems from children's magnitude comparisons (see Rousselle and Noël, 2007) as well as from differential effects of language characteristics on number line estimations (Helmreich et al., 2011). However, most of the studies so far that strived at examining the mental representation of magnitudes involved only symbolic estimations, which is in particular true for research with children (for an exception see Mejias et al., 2012). It might be worthwhile to directly compare the performance in symbolic and non-symbolic estimations and to relate it to the symbolic number knowledge. Sasanguie et al. (2012) have for instance found that children's performance in both a symbolic and a non-symbolic number line task were highly correlated but that only the symbolic task performance was associated with math performance–even if controlled for non-symbolic task performance (see also Sasanguie et al., 2013).

Furthermore, different *types of tasks* were used within symbolic estimations, such as position-to-number tasks (or perception tasks), where symbolic numbers have to be assigned to given non-symbolic magnitudes (e.g., Ashcraft and Moore, 2012) and number-to-position tasks (or production tasks), where non-symbolic magnitudes have to be generated that match given symbolic numbers (e.g., Barth and Paladino, 2011). Crollen et al. (2011) have shown that both tasks yield opposing biases (i.e., over-estimations in the production task and underestimations in the perception task) and different error rates in adults. A poorer performance in a production-like task compared to a perception-like task was also reported for children (Mundy and Gilmore, 2009; Mejias et al., 2012).

In addition, the *target stimuli* to be estimated differed. Continuous stimuli, such as in the number line paradigm (e.g., Siegler and Opfer, 2003), and discrete stimuli (e.g., numbers of dots, Crollen et al., 2011) have been used. Boyer et al. (2008) showed that children perform better in proportional judgments of liquids when they were presented as continuous amounts than by discrete units–probably as discrete magnitudes allured them to apply counterproductive counting mechanisms and suppressed a more intuitive approach. Moreover, children were also more accurate in comparing continuous (i.e., lengths of bars) than discrete magnitudes (i.e., numbers of dots; Barth et al., 2009).

Taken together, research so far has shown that the estimation type (i.e., symbolic vs. non-symbolic), task type (i.e., perception, production), and target type (continuous vs. discrete) might differently affect the shape and accuracy of magnitude estimations as well as the direction of the biases in terms of under- and overesti-mations. The next two issues refer to the variability and, again, to the shape of the estimations and, relatedly, to the inferred shape of the underlying mental representation of magnitudes.

Magnitude estimations can be *bounded* (e.g., number line tasks with lower and upper anchor points: Siegler and Opfer, 2003) or *unbounded* with no upper anchor cue (e.g., Booth and Siegler, 2006, Exp. 1; Whalen et al., 1999; Cohen and Blanc-Goldhammer, 2011). This issue is relevant in particular for the question of whether or not the estimations exhibit the signature of scalar variability. It seems likely that only unbounded tasks with no upper anchor cue would yield scalar variability as they do not allow for adjusting large estimations to an upper limit (Ebersbach et al., 2008). Furthermore, young children who lack an understanding of large numbers might fail to use the upper numerical anchor and their estimations thus might exhibit scalar variability, too, in cases where the upper anchor is unfamiliar.

Moreover, within the bounded number line tasks, many studies provided a lower and an upper anchor, such as 0 or 1 and 100 (e.g., Siegler and Opfer, 2003; Siegler and Booth, 2004; Booth and Siegler, 2006; Laski and Siegler, 2007; Opfer and Siegler, 2007; Ebersbach et al., 2008; Opfer and Thompson, 2008; Thompson and Opfer, 2008; Ashcraft and Moore, 2012; Ebersbach, in press), while in other studies the location of an *additional reference point* was explicitly referred to in the pre-test (e.g., the location of 50 on a number line of 0–100; Barth and Paladino, 2011; Bouwmeester and Verkoeijen, 2012; Slusser et al., 2013). The explicit indication of an additional reference point might have affected the shape of the estimations and facilitated the calibration of the estimations around the additional reference point. As a result, estimations might be best described by a cyclic power model with relative accurate estimations near the reference points, while the absence of a third reference point might rather yield a better fit with a logarithmic model.

## Methodological taxonomy

**Figure 1:**
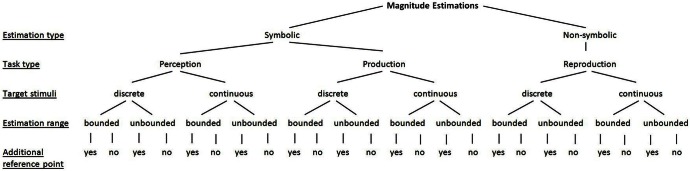
**Taxonomy of paradigms of studies on magnitude estimations**.

So far, we illustrated methodological differences between studies that might account for the often heterogeneous findings concerning the shape, variability, and accuracy of magnitude estimations. To solve this shortcoming, we propose a taxonomy into which each of the used paradigms might be classified (see Figure 1). This taxonomy accounts for (1) the question of whether symbolic numerals (or number words) are involved in the estimations or not (i.e., symbolic vs. non-symbolic estimations), (2) the type of the estimation tasks (i.e., perception, production, reproduction), (3) the type of the target stimuli (i.e., discrete vs. continuous), (4) the potential range of the estimations (i.e., bounded vs. unbounded), and (5) whether an additional reference point was provided or not. For instance, a classical number line paradigm (e.g., Siegler and Opfer, 2003) involves symbolic/numerical estimations in terms of a production task, in which the target stimuli are continuous, the estimation range is bounded by anchors and no additional reference point is provided.

## Suggestions for future research

First of all, it could be useful to systematically manipulate the methodological aspects proposed in the taxonomy in magnitude estimation tasks. This might allow determining if previous findings concerning the shape, variability, and accuracy of magnitude estimations and their underlying mental representation, as well as the emergence of systematic estimation skills in the course of development are generalizable or if they apply only to certain paradigms. If the latter was true, the different paradigms might tap different mental representations or, perhaps a certain paradigm might be no reliable and valid instrument to investigate the underlying mental representation (cf. Moeller and Nuerk, 2011).

Second, when aiming at identifying the mathematical model that explains magnitude estimations best, one has to take into account that model fits are affected, amongst others, by the number of trials, the number of parameters of the model, the question of whether a constant is estimated or not, and by the intra-individual variability of the estimations, which is relatively large in young children, in particular. One way to account for the model errors as well as for the number of free parameters would be using the Akaike information criterion (AIC), though it refers only to the relative fit of alternative models.

Third, shortcomings in deciding which model describes the shape of estimations of individual participants best should be prevented. Previous approaches largely differed, ranging from inferential statistics (i.e., comparing adjusted *R*^2^ values or the absolute values of the residuals of each model by *t*-tests: Siegler and Opfer, 2003; Moeller et al., 2009; Berteletti et al., 2012; though it cannot even be assumed that these parameters are Gaussian: May et al., 1989; Edgington and Onghena, 2007) to pure descriptive accounts (i.e., comparing *R*^2^ values or likelihoods of each model by visual inspection: Thompson and Opfer, 2008; Barth and Paladino, 2011; Cohen and Blanc-Goldhammer, 2011; Ashcraft and Moore, 2012). A descriptive approach provides maximally a heuristic but not a reliable decision rule (Glover and Dixon, 2004). Even slight differences between concurrent model fits might be overvalued if estimations were, for instance, classified as being linear only because the fit of a linear model is *R*^2^ = 0.731 and that of an alternative model is *R*^2^ = 0.730. In this regard, a statistically based classification method seems necessary to avoid arbitrary results (see also Moeller and Nuerk, 2011; Bouwmeester and Verkoeijen, 2012). In addition, if individual data will be analyzed in future research, a larger number of trials should be used to get more reliable data – although it also needs to be considered that a multitude of trials might reduce participants' motivation and yield interfering learning effects.

A fourth remark refers to the assumption, made implicitly or explicitly, that linear relationships between estimations and actual magnitudes, as reflected for instance by a better fit of a linear model (e.g., Siegler and Opfer, 2003), are the “idealized developmental endpoint of numerical estimation” (Ashcraft and Moore, 2012, p. 256; see also Hollands et al., 2002). Even if estimations rather obey a linear function, they might significantly and systematically deviate from the actual values, depending on the slope and intercept of the fitted linear function. Thus, even if equidis-tance between neighboring numbers is assumed, the estimations might deviate fundamentally from the actual values (cf. Moeller and Nuerk, 2011). In turn, estimations that are better explained by a power or logarithmic model might correspond on average better to the actual magnitudes than a linear model. However, the use of the best-fitting function might be questioned if the functions make similar predictions with respect to the observable estimation behavior (cf. Wagenaar, 1975; Dehaene, 2001; Thompson and Opfer, 2008; Cantlon et al., 2009). It thus might be useful to consider not only the shape of the estimations but also the accuracy in terms of both absolute and simple deviations from the actual values, as well as the variability of the estimations (see also Holloway and Ansari, 2008; White and Szucs, 2012).

Given the current state of research, future research might rather focus on conditions that lead to biased magnitude estimations and on how these estimations and the underlying “number sense” (Dehaene, 1997) might be improved. First attempts were already provided in the field of developmental research, where number games have proven to support equidistance in the mental representation of numbers (e.g., Wilson et al., 2006; Siegler and Ramani, 2008; Whyte and Bull, 2008). Other approaches might include promoting the familiarity with (e.g., Ebersbach et al., 2008) and the embodiment of numbers (e.g., Fischer et al., 2011) as potential precursors of an appropriate representation of the number system. Furthermore, the nature of estimation processes might be inspected further, such as when and how anchor cues are used or internally created–in particular in the course of development (see Schneider et al., 2008; Ashcraft and Moore, 2012; White and Szucs, 2012). It has been shown that adults use anchors to adjust their numerical estimations (Izard and Dehaene, 2008), but studies on whether children are able to do so and how their use of anchors might be affected (e.g., number knowledge, working memory) are rare (for exceptions see Newman and Berger, 1984; Petitto, 1990). Thus, the question of how and which estimation strategies are applied should be addressed. In addition, as strategies affect estimations (e.g., Ashcraft and Moore, 2012) one might question the fundamental assumption underlying the use of estimation paradigms, namely that estimations are a probate instrument to tap the underlying mental representation at all (Gescheider, 1988; Moeller and Nuerk, 2011). To sum up, we put forward a taxonomy that might contribute to a better comparability of studies on absolute magnitude estimations. We propose that the research focus might switch from trying to identify the model that describes the estimations best toward conditions and strategies that lead to estimation biases and toward procedures that might ward off these biases.

### Conflict of interest statement

The authors declare that the research was conducted in the absence of any commercial or financial relationships that could be construed as a potential conflict of interest.
